# Validation of the Turkish version of the ANDROTEST for detecting male hypogonadism in patients with sexual dysfunction

**DOI:** 10.1093/sexmed/qfag056

**Published:** 2026-08-17

**Authors:** Abdullah Akdag, Enes Aktas, Akif Erbin, Batu Akalin, Halil Lutfi Canat

**Affiliations:** University of Health Sciences, Department of Urology, Basaksehir Cam Sakura City Hospital, Istanbul, 34480, Turkey; University of Health Sciences, Department of Urology, Basaksehir Cam Sakura City Hospital, Istanbul, 34480, Turkey; University of Health Sciences, Department of Urology, Basaksehir Cam Sakura City Hospital, Istanbul, 34480, Turkey; University of Health Sciences, Department of Urology, Basaksehir Cam Sakura City Hospital, Istanbul, 34480, Turkey; University of Health Sciences, Department of Urology, Basaksehir Cam Sakura City Hospital, Istanbul, 34480, Turkey

**Keywords:** erectile dysfunction, hypogonadism, male, questionnaires, sensitivity and specificity

## Abstract

**Background:**

Male hypogonadism is an important endocrine cause of sexual dysfunction, yet symptom-based screening tools require cultural and linguistic validation to ensure their reliability across different populations.

**Aim:**

To evaluate the diagnostic performance, reliability, and validity of the Turkish version of the ANDROTEST in men presenting with sexual dysfunction.

**Methods:**

This prospective observational study included men aged 18-80 years presenting to the andrology outpatient clinic with symptoms of sexual dysfunction between February and November 2025. A control group consisted of age-matched individuals without clinical suspicion of hypogonadism or conditions affecting gonadal function. Participants completed the Turkish versions of Beck Depression Inventory (BDI) and the International Index of Erectile Function (IIEF). The ANDROTEST was translated and culturally adapted using a forward–backward translation process. Diagnostic performance was evaluated using receiver operating characteristic curve analysis, while test–retest reliability was assessed using the intraclass correlation coefficient (ICC). Multivariable logistic regression analysis was performed to identify independent predictors of hypogonadism.

**Outcomes:**

The primary outcome was the diagnostic performance of the Turkish ANDROTEST for identifying hypogonadism.

**Results:**

A total of 246 participants were included (120 controls and 126 patients with hypogonadism). Hypogonadal patients demonstrated significantly higher body mass index, triglycerides, total cholesterol, low-density lipoprotein cholesterol, and BDI, whereas total testosterone, free testosterone, sex hormone-binding globulin, high-density lipoprotein cholesterol, IIEF scores, and testicular volumes were significantly lower (all *P* < .05). The Turkish ANDROTEST demonstrated excellent diagnostic performance with an area under the curve of 0.980 (95% CI: 0.958-0.994). The optimal cut-off value of 11 yielded a sensitivity of 95.2% and specificity of 95.0%. In multivariable analysis, ANDROTEST remained an independent predictor of hypogonadism (adjusted OR 1.80, *P* = .009). Test–retest reliability was excellent (ICC = 0.99), and strong correlations were observed between the ANDROTEST score and IIEF scores, total testosterone levels, and depression scores (all *P* < .001).

**Clinical Implications:**

The Turkish version of the ANDROTEST may serve as a practical screening tool for identifying men at risk of hypogonadism in routine clinical practice.

**Strengths and Limitations:**

Strengths include the prospective design and comprehensive evaluation of psychometric and diagnostic performance. Limitations include the single-center design and the case–control structure, which may overestimate diagnostic performance.

**Conclusion:**

The Turkish version of the ANDROTEST demonstrated excellent diagnostic performance and strong reliability for identifying male hypogonadism in men with sexual dysfunction, supporting its potential utility as a culturally adapted screening tool in clinical practice.

## Introduction

Sexual dysfunction is a common problem for men, but the rates of this problem vary between different groups of people and age groups. A recent study found that 24.2% of US men had the condition, with higher rates in older groups: 48.0% of men aged 65 to 74 and 52.2% of men aged 75 and older.^1^ General health status, lifestyle factors, and the presence of comorbid conditions may significantly influence the prevalence of sexual dysfunction. Psychological factors such as stress and anxiety, along with socioeconomic status and access to healthcare, may also contribute to its development and clinical presentation. Among the various biological factors implicated in male sexual dysfunction, hypogonadism represents an important and potentially treatable endocrine cause.^2^

The ANDROTEST is a systematic interview intended to assess male hypogonadism in individuals experiencing sexual dysfunction. This 12-item instrument has been effective in detecting androgen insufficiency, facilitating timely intervention.^3^ Cultural and linguistic disparities can affect patients’ perceptions and reporting of symptoms, highlighting the necessity for validation studies of these tools across varied groups. In this context, in a different country, socio-cultural factors may influence the expression and recognition of sexual health issues. In this case, if a standardized scoring method tailored to that language is not employed, disparate and erroneous outcomes may arise.^4^

A population-based survey conducted in Turkey reported that the prevalence of erectile dysfunction among men aged ≥40 years is approximately 33%, with age identified as the primary predictor of both its presence and severity.^5^ Despite the relatively high prevalence of sexual dysfunction in this population, validated tools specifically adapted to the Turkish language for the assessment of androgen deficiency remain limited. Moreover, the ANDROTEST has previously been validated primarily in its original population, and data regarding its performance in different cultural settings are scarce.

Therefore, the aim of the present study was to evaluate the psychometric properties, reliability, and validity of the Turkish version of the ANDROTEST in men presenting with sexual dysfunction. By providing a culturally adapted and validated diagnostic instrument, this study may contribute to improving the clinical identification of androgen deficiency in Turkish-speaking patients. In addition, the findings may offer further external validation of the ANDROTEST and support its broader applicability across different sociocultural contexts.

## Methods

### Compliance with ethical standards

The present study received approval from the Institutional Medical Ethics Committee and commenced after the ethics committee approval, and verbal and written consent were obtained from all patients (Approval no. 2025-28, date: 30.01.2025).

### Study design and data collection

Male patients aged 18-80 years who presented to the andrology outpatient clinic with symptoms of sexual dysfunction between February and November 2025 were prospectively assessed for eligibility. Among these patients, those who fulfilled the clinical and biochemical criteria for hypogonadism were included in the hypogonadism group. Hypogonadism was defined as the presence of symptoms compatible with androgen deficiency together with low morning total testosterone confirmed on two separate measurements.

The inclusion criteria for the hypogonadism group were age between 18 and 80 years, presentation with sexual dysfunction symptoms, ability to understand and respond to the structured interview in Turkish, and provision of written informed consent. Patients were excluded if they had insufficient Turkish language proficiency, inability to provide informed consent, inability to understand the interview items or provide reliable responses because of clinically evident cognitive impairment, previous or current testosterone therapy, acute systemic illness, or medication use or endocrine conditions that could substantially interfere with gonadal function unless already accounted for in the diagnostic evaluation. Cognitive eligibility was assessed clinically by the evaluating physician during the initial face-to-face assessment and was not evaluated using a formal neuropsychological test.

**Figure 1:**
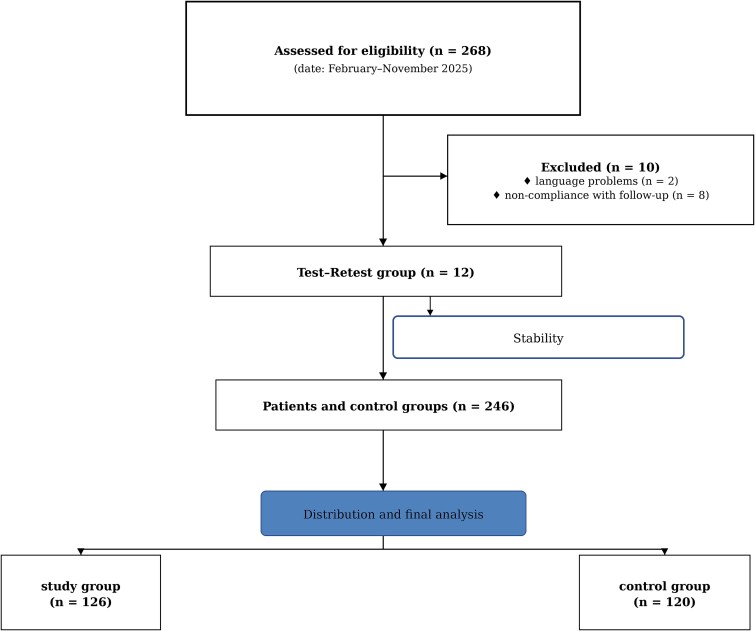
Flowchart of the study.

The comparison group consisted of consecutive men attending the urology outpatient clinic for non–androgen-related complaints during the same study period, who had no clinical suspicion of androgen deficiency and testosterone values above the diagnostic threshold on two separate morning measurements. Before inclusion, all potential controls underwent clinical and biochemical screening to exclude overt or subclinical androgen deficiency. This screening included assessment of symptoms suggestive of hypogonadism, review of medical history, medication use, endocrine disorders, and comorbid conditions potentially affecting gonadal function. Individuals with clinical suspicion of androgen deficiency, total testosterone values below the diagnostic threshold on either measurement, or conditions/medications that could influence gonadal function were excluded from the control group (Figure 1).

The age, body mass index (BMI), and testicular volume (assessed using ultrasound) of all patients were prospectively recorded. Laboratory analyses encompassed blood glucose, total cholesterol, high-density lipoprotein (HDL) cholesterol, and triglycerides. The results of total testosterone, free testosterone, prolactin, luteinizing hormone (LH), follicle-stimulating hormone (FSH), and thyroid-stimulating hormone (TSH) were also recorded. When values were borderline, confirmation with repeat testing was performed. Blood samples were obtained in the morning between 9:00 and 10:00 AM following an overnight fast.

Additionally, patients completed the Turkish-validated versions of the Beck Depression Inventory and the International Index of Erectile Function (IIEF) questionnaires.^6^^,^^7^ The Turkish ANDROTEST was administered as a face-to-face structured interview by two male physicians experienced in andrology/urology practice. Before study initiation, both physicians were instructed on the standardized wording, scoring procedure, and administration of the Turkish ANDROTEST to ensure consistency. For the test–retest reliability assessment, the second interview was performed two weeks later by the same physician who conducted the first interview, before the initiation of any treatment. The first ANDROTEST administration was used for all primary analyses, including diagnostic performance assessment, receiver operating characteristic (ROC) curve analysis, convergent validity analyses, and logistic regression models. The second ANDROTEST administration, performed two weeks later by the same physician before the initiation of any treatment, was used only for test–retest reliability assessment and calculation of the intraclass correlation coefficient (ICC).

### Diagnosis of hypogonadism

Hypogonadism was defined according to the EAU guidelines as the presence of clinical symptoms of androgen deficiency together with a total testosterone level < 3.5 ng/mL (12 nmol/L).^8^ Blood samples were obtained in the morning between 9:00 and 10:00 AM after an overnight fast. Total testosterone was measured twice in all participants on separate morning samples before final classification. Participants were classified according to the repeated testosterone measurements together with clinical assessment.

### Translation process and pilot testing

The validation study of the ANDROTEST form in Turkish was carried out with electronic consent from the original authors of the form. Two different expert translators translated the original English text into Turkish independently (official, signed, and sealed document available). The researchers, including a highly experienced academician in the field of andrology (HLC), examined the translations and finalized the Turkish version. The completed document was subsequently back-translated into English by a different professional translator. The discrepancies between the original version and the back-translated iteration were assessed, and requisite amendments were implemented. Following this process, a preliminary study was undertaken with 15 participants. In this study, following the completion of the forms, patients were asked about which questions they found challenging to comprehend. In light of this feedback, minor amendments were implemented, resulting in the final Turkish version of the form (Supplementary File).

### Sample size

Methodological guidelines and previous psychometric research generally recommend a sample size of at least 5-10 people per item for validation studies.^9^ Considering that the ANDROTEST comprises 12 items, a minimum of 120 participants was deemed enough for comprehensive factor analysis and reliability assessment. Consequently, the final study population consisted of two groups: 126 patients with clinically and biochemically defined hypogonadism and 120 controls without clinical or biochemical evidence of androgen deficiency. Test–retest reliability was evaluated in a pilot subgroup of patients who completed the ANDROTEST twice with a two-week interval. This approach was adopted to minimize patient burden while ensuring that the second assessment was performed before the initiation of any treatment that might influence symptom reporting. No therapeutic intervention was introduced between the two administrations. This approach was consistent with previous validation studies in the literature.^10^

### Statistical analysis

The statistical analysis of the study was performed using Python version 3.13 with the libraries pandas, numpy, scipy, statsmodels, scikit-learn, and matplotlib. The normality of the variables was evaluated by histogram visuals, coefficient of variation tables, skewness and kurtosis metrics, normal Q-Q plots, detrended normal Q-Q plots, and the Kolmogorov–Smirnov test. In the Kolmogorov–Smirnov test, a non-significant *P*-value (*P* > .05) was deemed the principal criterion for evaluating normal distribution. If at least two of the remaining four criteria indicated normality, the data were considered normally distributed. Data that followed a normal distribution were presented as mean ± standard deviation, while data that did not follow a normal distribution were presented as median values and interquartile ranges. Categorical variables were presented as frequency and percentages (n, %). Numerical variables that exhibited a normal distribution across two independent groups were assessed using the independent samples *t-*test, whereas those deviating from normality were evaluated with the Mann–Whitney U test. Relationships among categorical variables were analyzed using the chi-square test, and when more than 20% of the expected cell counts were below 5, Fisher’s exact test was applied instead.

Diagnostic performance of the ANDROTEST for identifying hypogonadism was evaluated using ROC curve analysis with calculation of area under the curve (AUC), 95% confidence intervals, optimal cut-off values based on the Youden index, sensitivity, specificity, positive and negative predictive values, accuracy, and generation of confusion matrices for selected thresholds. Univariate logistic regression analyses were performed to ascertain associations between clinical variables and hypogonadism. Variables with *P* < .10 were subsequently incorporated into a multivariable logistic regression model to identify independent predictors, with results presented as odds ratios and 95% confidence intervals. Multicollinearity among predictors was assessed using variance inflation factor (VIF) analysis. VIF values greater than 10 were considered indicative of substantial multicollinearity. We used ICC analysis to see how reliable the ANDROTEST was when given to the same people twice. Convergent validity was assessed via Spearman correlation analyses between the ANDROTEST score and pertinent hormonal and clinical parameters. Because ANDROTEST is a structured clinical interview composed of heterogeneous symptom domains rather than a psychometric scale measuring a single latent construct, exploratory factor analysis was not considered necessary. For all analyses, a two-tailed *P*-value of less than .05 was considered statistically significant.

## Results

A total of 246 participants were included in the final analysis, comprising 120 individuals in the control group and 126 patients in the hypogonadism group. Baseline demographic and clinical characteristics of the study population are summarized in Table 1. Age did not differ significantly between groups (*P* = .243). Patients with hypogonadism had significantly higher body weight, BMI, triglyceride levels, total cholesterol, low-density lipoprotein cholesterol, and Beck Depression Inventory scores compared with controls. In contrast, total testosterone, free testosterone, sex hormone-binding globulin (SHBG), HDL cholesterol, IIEF total score, IIEF sexual desire score, and IIEF sexual satisfaction score were significantly lower in the hypogonadism group (all *P* < .05). Testicular volumes were also significantly reduced among hypogonadal patients.

**Table 1:** Baseline demographic and clinical characteristics of the study population.

Variable	Control (*n* = 120)	Hypogonadism (*n* = 126)	*P*-value
Age (years)	45.49 ± 12.20	47.21 ± 10.82	.243^a^
Body weight (kg)	80.00 [75.00-88.00]	85.00 [78.00-95.00]	** .001** ^**b**^
Height (m)	1.76 [1.70-1.79]	1.75 [1.70-1.78]	.069^b^
BMI (kg/m^2^)	26.55 [24.81-28.48]	28.40 [25.85-31.65]	**<.001** ^**b**^
Right testis volume (mL)	19.00 [16.00-21.00]	18.00 [16.00-20.00]	**<.001** ^**b**^
Left testis volume (mL)	17.50 [16.00-20.00]	16.00 [14.00-18.00]	**<.001** ^**b**^
Total testis volume (mL)	36.68 ± 5.56	33.14 ± 6.19	**<.001** ^a^
LH (mIU/mL)	6.96 [5.29-8.50]	5.93 [4.50-8.96]	.147^b^
FSH (mIU/mL)	5.77 [3.78-7.41]	5.83 [3.78-9.55]	.175^b^
Prolactin (ng/mL)	9.17 [7.33-11.60]	8.66 [6.32-12.38]	.495^b^
SHBG (nmol/L)	34.90 [24.88-45.47]	19.30 [15.00-27.52]	**<.001** ^**b**^
Albumin (g/dL)	47.00 [45.00-49.00]	46.00 [44.00-48.00]	.028^b^
Total testosterone (ng/mL)	4.93 [4.20-6.45]	2.49 [1.98-3.00]	**<.001** ^**b**^
Free testosterone (ng/dL)	18.58 [15.37-26.00]	9.73 [7.05-12.40]	**<.001** ^**b**^
Triglycerides (mg/dL)	112.00 [84.00-165.00]	167.00 [120.25-266.50]	**<.001** ^**b**^
HDL cholesterol (mg/dL)	44.16 ± 11.55	39.27 ± 8.27	**<.001** ^a^
LDL cholesterol (mg/dL)	100.00 [81.00-125.00]	118.50 [88.00-136.75]	** .016** ^**b**^
Total cholesterol (mg/dL)	175.77 ± 36.47	197.32 ± 44.11	**<.001** ^a^
TSH (μIU/mL)	1.60 [1.24-2.18]	1.65 [1.09-2.26]	.462^b^
Beck Depression Inventory	5.00 [3.75-8.00]	20.00 [16.00-24.00]	**<.001** ^**b**^
IIEF sexual desire score	8.00 [8.00-9.00]	5.00 [4.00-6.00]	**<.001** ^**b**^
IIEF sexual satisfaction score	12.00 [11.00-14.00]	7.00 [6.00-8.00]	**<.001** ^**b**^
IIEF total score	66.00 [63.00-70.25]	36.00 [32.00-42.00]	**<.001** ^**b**^
ANDROTEST score	5.00 [3.00-6.25]	16.00 [15.00-18.00]	**<.001** ^**b**^
COVID-19 history	56 (46.7%)	63 (50.0%)	.693^c^
Diabetes mellitus	32 (26.7%)	49 (38.9%)	.057^c^
Regular partner	118 (98.3%)	121 (96.0%)	.447^c^
Cryptorchidism	0 (0.0%)	12 (9.5%)	** .002** ^**c**^

Abbreviations: BMI = body mass index; FSH = follicle-stimulating hormone; HDL = high-density lipoprotein; IIEF = International Index of Erectile Function; IQR = interquartile range; LDL = low-density lipoprotein; LH = luteinizing hormone; SD = standard deviation; SHBG = sex hormone-binding globulin; TSH = thyroid-stimulating hormone.

^a^Student’s *t*-test ^b^Mann–Whitney U test ^c^Chi-square

**Figure 2:**
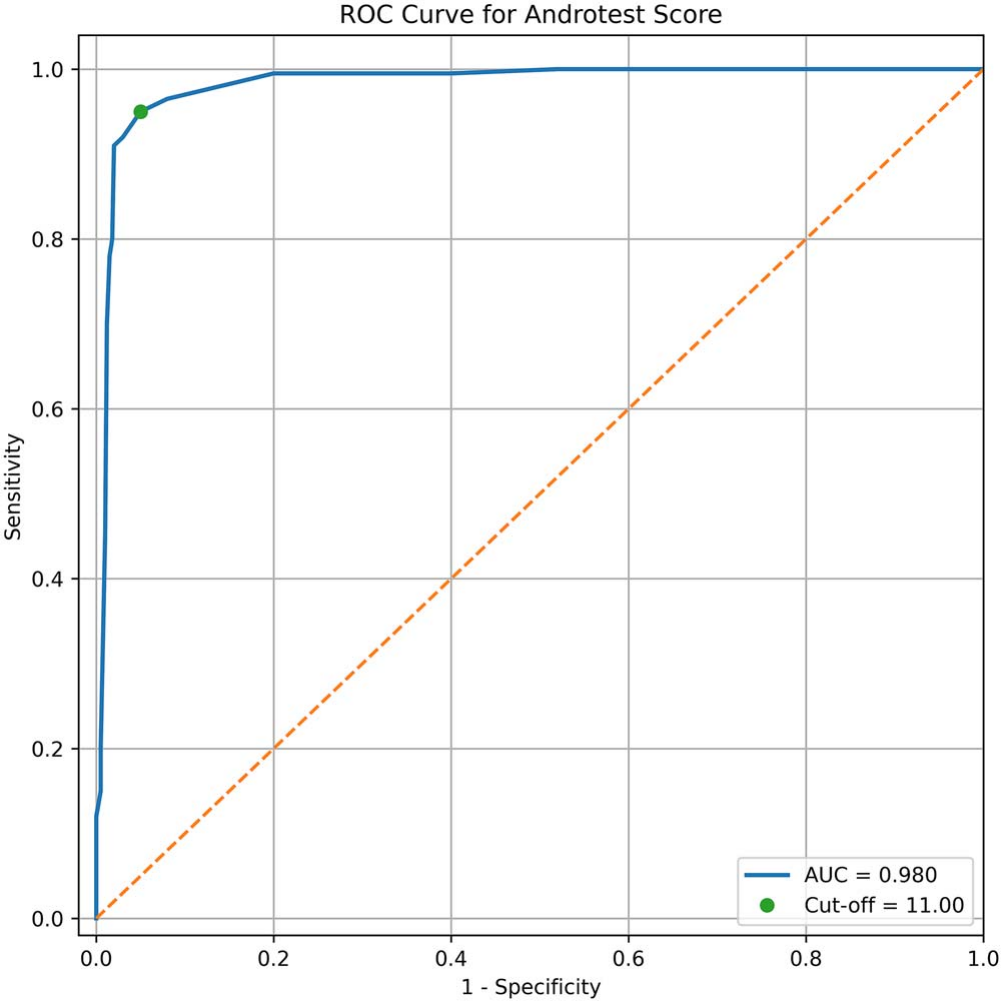
ROC curve analysis of the Turkish ANDROTEST.

ROC curve analysis (Figure 2) was performed to evaluate the diagnostic performance of the Turkish ANDROTEST for identifying hypogonadism. The AUC was 0.980 (95% CI: 0.958-0.994), indicating high discriminatory performance between groups. The Youden index found that the best at a different cutoff value was 11. This gave a sensitivity of 95.2%, a specificity of 95.0%, a positive predictive value of 95.2%, a negative predictive value of 95.0%, and an overall accuracy of 95.1%. At an alternative cut-off value of 8, sensitivity increased to 99.2%, whereas specificity decreased to 80.0%, with an overall accuracy of 89.8% (Table 2).

**Table 2:** Diagnostic performance of the Turkish ANDROTEST at different cut-off values.

Cut-off value	Sensitivity	Specificity	PPV	NPV	Accuracy
8	0.992	0.800	0.839	0.990	0.898
11 (Youden Index)	0.952	0.950	0.952	0.950	0.951

Abbreviations: NPV = negative predictive value; PPV = positive predictive value.

Univariable logistic regression analyses were performed in the entire study population of 246 participants, comprising 126 hypogonadal patients and 120 controls (Table 3). The dependent variable was binary hypogonadism status, defined by the presence of androgen-deficiency symptoms together with total testosterone <3.5 ng/mL confirmed on two separate morning measurements. Significant predictors included the ANDROTEST score, SHBG, Beck Depression Index, IIEF sexual desire, triglyceride levels, total testis volume, BMI, HDL cholesterol, FSH and the presence of diabetes mellitus (all *P* < .05). Variables with *P* < .10 in univariate analysis were entered into a multivariable logistic regression model. No significant multicollinearity was observed, with all VIF values below 10. In the multivariable model, the ANDROTEST score (adjusted OR 1.80, 95% CI 1.16-2.78, *P* = .009), SHBG (adjusted OR 0.77, 95% CI 0.61-0.96, *P* = .020), Beck Depression Index (adjusted OR 1.85, 95% CI 1.09-3.13, *P* = .022), and FSH levels (adjusted OR 1.38, 95% CI 1.02-1.86, *P* = .036) remained independent predictors of hypogonadism.

**Table 3:** Univariate and multivariable logistic regression analyses for predictors of hypogonadism.

Variable	Univariate OR (95% CI)	*P*-value	Adjusted OR (95% CI)	*P*-value
ANDROTEST score	2.01 (1.68-2.40)	<.001	**1.80 (1.16**-**2.78)**	**.009**
SHBG	0.91 (0.89-0.94)	<.001	**0.77 (0.61**-**0.96)**	**.020**
Beck depression index	1.69 (1.47-1.93)	<.001	**1.85 (1.09**-**3.13)**	**.022**
IIEF sexual desire	0.14 (0.09-0.23)	<.001	0.57 (0.17-1.96)	.372
Triglycerides	1.01 (1.00-1.01)	<.001	1.02 (0.99-1.04)	.060
Total testis volume	0.90 (0.86-0.94)	<.001	0.88 (0.70-1.12)	.309
BMI	1.14 (1.07-1.23)	<.001	0.12 (0.0003-51.85)	.489
HDL cholesterol	0.95 (0.93-0.98)	<.001	1.05 (0.88-1.26)	.568
FSH	1.06 (1.01-1.11)	.011	**1.38 (1.02**-**1.86)**	**.036**
Diabetes mellitus	1.75 (1.02-3.00)	.042	0.42 (0.03-6.03)	.520
TSH	0.82 (0.62-1.09)	.173	–	–
Age	1.01 (0.99-1.04)	.241	–	–
LH	1.03 (0.98-1.08)	.243	–	–
Prolactin	1.02 (0.99-1.05)	.270	–	–

Abbreviations: BMI = body mass index; CI = confidence interval; FSH = follicle-stimulating hormone; HDL = high-density lipoprotein; IIEF = International Index of Erectile Function; LH = luteinizing hormone; OR = odds ratio; SHBG = sex hormone-binding globulin; TSH = thyroid-stimulating hormone.

Test–retest reliability of the Turkish ANDROTEST was evaluated in a subgroup of patients who completed the questionnaire twice with a two-week interval. The ICC for the total ANDROTEST score was 0.99 (95% CI: 0.96-1.00), demonstrating excellent temporal stability and reproducibility (Table 4).

**Table 4:** Test–retest reliability of the Turkish ANDROTEST.

ICC Type	ICC	95% CI
ICC^1^^,^^2^	**0.99**	**0.96-1.00**

Abbreviation: ICC = Intraclass Correlation Coefficient.

Spearman correlation analysis (Table 5) was performed to assess the convergent validity of the ANDROTEST. The ANDROTEST exhibited significant negative correlations with the IIEF total score (r = –0.887), IIEF sexual satisfaction (r = –0.868), IIEF sexual desire (r = –0.838), and total testosterone (r = –0.743) (all *P* < .001). There was a strong positive relationship with the Beck Depression Index (r = 0.850, *P* < .001). Moderate correlations were observed with free testosterone, SHBG, BMI, and triglyceride levels. These results substantiate the construct validity of the Turkish ANDROTEST in evaluating symptoms associated with hypogonadism and sexual dysfunction.

**Table 5:** Convergent validity analysis of the Turkish ANDROTEST.

Variable	Spearman r	*P*-value
IIEF total score	**–0.887**	<.001
IIEF sexual satisfaction	**–0.868**	<.001
IIEF sexual desire	**–0.838**	<.001
Beck Depression Index	** 0.850**	<.001
Total testosterone	**–0.743**	<.001
Free testosterone	**–0.639**	<.001
SHBG	–0.392	<.001
Triglycerides	0.309	<.001
BMI	0.306	<.001
HDL cholesterol	–0.138	.031
LDL cholesterol	0.107	.095
FSH	0.099	.121
LH	–0.101	.114

Abbreviations: BMI = body mass index; FSH = follicle-stimulating hormone; HDL = high-density lipoprotein; IIEF = International Index of Erectile Function; LDL = low-density lipoprotein; LH = luteinizing hormone; SHBG = sex hormone-binding globulin.

## Discussion

In this study, the Turkish version of the ANDROTEST demonstrated strong reliability and validity for the structured assessment of hypogonadism-related symptoms in men presenting with sexual dysfunction. The instrument showed high discriminatory performance in this validation cohort, achieving an AUC of 0.980 and optimal sensitivity and specificity exceeding 95% at the Youden-derived cut-off value of 11. Hypogonadal patients demonstrated markedly elevated metabolic risk parameters and depressive symptoms, coupled with diminished testosterone levels, IIEF scores, and testicular volumes, thereby affirming the clinical coherence of the cohort. Additionally, the ANDROTEST persisted as an independent predictor of hypogonadism in multivariable analysis, and its test–retest reliability was outstanding (ICC = 0.99). These findings support the clinical and psychometric validity of the Turkish adaptation within the context of the present validation cohort. However, the magnitude of the observed diagnostic performance should be interpreted cautiously in light of the study design and patient-selection strategy.

Previous reports have highlighted the limitations of currently available screening questionnaires for hypogonadism. While validated instruments such as ADAM, qADAM, AMS, MMAS, and ANDROTEST are commonly cited, their diagnostic performance varies considerably, with sensitivities and specificities that make them suboptimal as stand-alone screening tools or surrogates for biochemical testing.^11^ In a systematic review, Millar et al. emphasized that among all symptom-based tools, ANDROTEST demonstrated the most favorable balance of diagnostic accuracy.^4^ Nevertheless, both studies underscored an important limitation: ANDROTEST had only been validated in its original Italian population, without cross-cultural adaptation or external validation in other languages. Against this backdrop, the present study represents the first effort to translate and validate the ANDROTEST in a different language and patient population. By addressing this gap, our findings contribute to the generalizability of the tool and support its potential broader use in diverse clinical settings.

From a clinical perspective, the ANDROTEST provides an important contribution to the diagnostic process of late-onset hypogonadism by enabling a faster, more accurate, and standardized assessment of symptom domains.^12^ Unlike unstructured history-taking, the structured nature of the instrument reduces inter-observer variability and promotes consistency across different evaluators, which is particularly valuable in both clinical practice and research settings. Furthermore, its systematic format facilitates the training of clinicians and enhances adherence to a standardized approach in evaluating suspected cases of androgen deficiency.^12^ In this regard, the use of a validated version of the ANDROTEST in diverse populations not only strengthens diagnostic reliability but also supports the development of harmonized clinical pathways for the management of symptomatic hypogonadism.

Although the present design does not fully reflect the original intended-use population of the ANDROTEST, which was developed to identify hypogonadism among men presenting with sexual dysfunction, the primary aim of this study was to provide the first Turkish linguistic and cultural validation of the instrument. In this context, the recruitment of the comparison group from general urology practice may also offer preliminary, hypothesis-generating evidence that structured symptom assessment could be relevant beyond highly specialized sexual medicine clinics. This may be particularly important for late-onset hypogonadism, which is increasingly encountered in general urological practice and may remain underrecognized because of nonspecific and overlapping symptoms. Although contemporary guidelines do not support symptom-based questionnaires as diagnostic substitutes for biochemical testing, structured interviews may still have practical value in standardizing symptom assessment, raising clinical suspicion, and identifying patients who require hormonal evaluation.^8^ Therefore, the Turkish ANDROTEST should not be interpreted as a replacement for testosterone measurement, but rather as a structured screening aid that may help guide biochemical assessment in appropriate clinical contexts. Further studies in unselected general urology and sexual dysfunction cohorts are needed to confirm this broader applicability.

The current results further substantiate the potential clinical utility of the Turkish ANDROTEST by illustrating high discriminatory ability for hypogonadism in this cohort. Nevertheless, the very high diagnostic accuracy observed in the present study should not be interpreted as definitive evidence of real-world screening performance. The case–control design, which compared clinically distinct hypogonadal and eugonadal individuals, may have introduced spectrum bias and inflated the apparent discriminatory performance compared with less selected clinical or primary-care populations. The Youden index found that the best cut-off value was 11, which gave a favorable balance between sensitivity and specificity. We also looked at the original cut-off value of >8 to make sure that our methods were similar to those used in the first ANDROTEST study.^3^ At this lower cut-off, sensitivity rose significantly while specificity fell, suggesting that a threshold of 8 may be better for screening settings where reducing false negatives is most important. On the other hand, a cut-off of 11 seems better for clinical decision-making that needs more precise diagnosis. Accordingly, the cut-off value identified in this study should be regarded as an initial estimate for the Turkish version rather than as a universally applicable threshold. Further prospective studies in broader, less selected cohorts are required to determine whether the same threshold maintains similar diagnostic performance in routine clinical practice.

The robust negative correlations between IIEF scores and total testosterone, alongside the positive correlation with depressive symptoms, indicate that the scale reflects the multifaceted clinical presentations of hypogonadism rather than mere biochemical changes.^13^ However, because the ANDROTEST is a structured symptom-based interview, several of its domains overlap with the clinical manifestations used to suspect or define male hypogonadism. This overlap raises the possibility of incorporation bias, which may partly explain both the strong association observed in multivariable analysis and the high ROC-derived diagnostic performance. Therefore, the independent predictive value of ANDROTEST should be interpreted in the context of a symptom-based screening instrument rather than as evidence that it is a stand-alone diagnostic marker. Biochemical confirmation with morning testosterone measurement remains necessary for the diagnosis of hypogonadism and for treatment decisions. In addition, the high test–retest reliability supports the temporal stability of the Turkish ANDROTEST and indicates that the structured interview yields reproducible scores over a short interval before treatment initiation. Overall, these results show that the Turkish version preserves the conceptual framework of the original ANDROTEST while providing a culturally adapted structured screening aid for Turkish-speaking men presenting with sexual dysfunction.

This study has certain limitations. First, the single-center design and the inclusion of only men presenting to a specialized andrology outpatient clinic with sexual dysfunction limit the external validity of the findings. Therefore, the present results should primarily be interpreted within this clinical context and may not be directly generalizable to community-based populations, primary-care settings, asymptomatic men, or men presenting with non-sexual complaints. Second, while validation studies typically juxtapose hypogonadal and eugonadal cohorts, the etiological diversity of hypogonadism—stemming from various genetic and endocrine disorders—indicates that the construct validity of a standardized questionnaire may only partially reflect the clinical spectrum. Moreover, confining the design to two extreme groups (hypogonadal vs. eugonadal) is methodologically constrained; the incorporation of an intermediate “borderline” cohort would more accurately represent real-world practice and facilitate a more precise evaluation of discriminative validity and generalizability. Therefore, the present design does not fully reflect the intended clinical use of the ANDROTEST, which is to identify hypogonadism among men with sexual dysfunction. This may have increased group separation and contributed to the high diagnostic accuracy and higher optimal cut-off observed in this study. Although controls were screened clinically and biochemically to reduce the possibility of unrecognized or subclinical hypogonadism, this careful selection may have increased group separation and may have contributed to the high diagnostic performance observed in ROC analysis. Third, the sample size, while sufficient for validation analyses, may not comprehensively represent the heterogeneity of the larger population. Fourth, as discussed above, the symptom-based structure of ANDROTEST may introduce incorporation bias because some item domains overlap with the clinical criteria used to suspect hypogonadism. For this reason, the reported associations and ROC-derived indices should be interpreted as the performance of a structured screening aid within a controlled validation framework, not as a substitute for hormonal diagnosis. Despite these limitations, the current study is the first to assess the reliability and validity of ANDROTEST in Turkish. Its prospective design, incorporation of both initial administration and test–retest analysis, and thorough evaluation of psychometric properties enhance the validity of our findings and underscore the clinical significance of a culturally adapted and standardized instrument for hypogonadism screening. Future multicenter studies including broader, less selected, and mixed-risk Turkish-speaking populations are required to confirm external validity, refine the optimal cut-off value, and determine the real-world screening performance of the Turkish ANDROTEST.

## Conclusion

The Turkish version of the ANDROTEST is a reliable and culturally adapted structured interview for assessing hypogonadism-related symptoms in men presenting with sexual dysfunction. In this initial single-center validation cohort, it demonstrated strong test–retest reliability and high discriminatory performance for identifying clinically and biochemically defined hypogonadism. However, because of the case–control design, careful control selection, and symptom overlap with diagnostic criteria, its diagnostic accuracy should be interpreted cautiously. The Turkish ANDROTEST should therefore be considered a structured screening aid to identify men who may require biochemical testosterone evaluation, rather than a stand-alone diagnostic tool. Further multicenter studies in broader, less selected populations are required to confirm external validity, refine cut-off values, and assess its prognostic relevance.

## Supplementary Material

ANDROTEST-Turkce_qfag056

## Data Availability

The dataset is not publicly available but may be accessed upon reasonable request and with appropriate authorization from the relevant authorities.
