# Supplementary material for: Validation of the Turkish version of the ANDROTEST for detecting male hypogonadism in patients with sexual dysfunction
Source: Sex Med. 2026 Aug 17;14(5):qfag056. doi: 10.1093/sexmed/qfag056 (PMC13480882; doi:10.1093/sexmed/qfag056)
Supplement: Supplementary file 1 [file androtest-turkce_qfag056.pdf]

Ek: ANDROTEST Uygulama Talimatları

ANDROTEST hipogonadizmin (cinsel işlev bozukluğu ile başvuran bireylerde 12nmol/L veya 3.5ng/mL'nin altındaki total testosteron seviyeleri olarak tanımlanır) değerlendirilmesi için tasarlanmış yapılandırılmış bir görüşmeden oluşur. Bu değerlendirme yalnızca son 3 ay içinde en az bir kez cinsel ilişkiye girdiğini bildiren hastalar için geçerlidir. Görüşme 12 temel sorudan oluşmaktadır. Muayene eden kişi, koyu renkle vurgulanan soruları, belirtilen ifadelere sıkı sıkıya bağlı kalarak kelimesi kelimesine sormalıdır. Gerekirse hastanın yanıtlarını netleştirmek için normal metin içinde sunulan ek sorulardan yararlanılabilir. Hastanın cevaplarını kendi kelimeleriyle ifade ederek özgürce yanıt vermesine izin verilmelidir. Muayene eden kişi, her sorudan sonra verilen ayrıntılı puanlama kriterlerine dayanarak hastanın yanıtlarını 0 ila 3 arasında bir ölçekte kodlamaktan sorumludur. Bazı sorular evet/hayır şeklinde ikili bir yanıt gerektirmektedir. Soruların sunulma sırası kesinlikle korunmalıdır, çünkü belirlenen sıralamadan herhangi bir sapma hastanın yanıtlarını potansiyel olarak etkileyebilir.

1) Yaş *Hastaya yaşını sorduktan sonra, hastanın yaşına göre uygun bir puan verin.*

0 <40 yaş

1 40–49 yaş

2 50–59 yaş

3 >59 yaş

2) Cinsel gelişiminizi (ergenlik) ne zaman geçirdiniz?

Cinsel gelişiminizi hangi yaşta geçirdiniz? Ergenliği okul arkadaşlarınızla aynı zamanda mı yaşadınız? Kasık kılları ve genital gelişimin sizde ve okul arkadaşlarınızda aynı anda olup olmadığını fark ettiniz mi?

Hastanın, cinsel gelişimi okul arkadaşlarıyla aynı zamanda veya onlardan önce yaşadığını bildirmesi durumunda 0 puan verin; ergenliğin gecikmiş olduğunu belirten hastalar için 3 puan verin

0 9–14 yaş (normal)

3 >14 yaş (gecikmiş)

3) Hiç hipofiz hastalığınız oldu mu?

Hiç hipofiz hastalığı nedeniyle ameliyat oldunuz mu? Hiç hipofiz hastalığı için tıbbi tedavi gördünüz mü? *Hastalar hipofiz hastalığını bildirmediyse 0 puan, evet cevabı verdiyse 3 puan verin.*

0 No

3 Yes

4) Hiç inmemiş testis (kriptorşidizm) tanısı aldınız mı?

Kriptorşidizm için hiç cerrahi müdahale geçirdiniz mi? Kriptorşidizm için hiç tıbbi tedavi aldınız mı?

Hasta kriptorşidizm öyküsü bildirmezse (tek taraflı vakalar dahil) 0 puan verilirken, olumlu yanıt için 3 puan verilecektir.

0 Hayır

3 Evet

5) Lütfen cinsel ilişki sırasındaki deneyiminizi tarif edin: ereksiyona ulaşmakta ne sıklıkla zorluk yaşıyorsunuz?

Sorunun tanımı son 3 ayı kapsamaktadır. Bazen = <%25 Sık = %25–49, Oldukça sık = %50–74, Her zaman = >%75 olguda.

0 Bazen

1 Sık

2 Oldukça Sık

3 Her zaman

6) Hiç ereksiyon ile uyanıyor musunuz?

Bu durum son 3 ay içinde ne sıklıkla meydana geldi? *Hasta spontan gece veya sabah ereksiyonlarını daha önce gözlemlediği sıklıkta bildiriyorsa 0 puan verilir. Bu tür ereksiyonlar mevcutsa ancak son üç ay içinde sıklıkları bir miktar azalmışsa 1 puan verilir. Sıklıkları en az %50 azalmışsa 2 puan verilir. Gece veya sabah ereksiyonları görülmüyorsa 3 puan verilir.*

0 Evet, düzenli olarak

1 Geçmişte olduğundan daha az

2 Sadece arada bir

3 Hiçbir zaman

7) Son üç ay içinde ne sıklıkla otoerotik aktivitede (mastürbasyon) bulundunuz?

0 >8 kez/ay

1 3–7 kez/ay

2 1–2 kez/ay

3 Hiçbir zaman

Eğer hasta otoerotizm yapmıyorsa (cevap 3), bir sonraki soru (#8) geçerli değildir. Bu durumda soru 8'e 1 puan verin ve soru #9'a geçin.

8) Otoerotizm (mastürbasyon) sırasında nasıl hissediyorsunuz?

Yukarıdaki soruyu sorduktan sonra şu puanlamayı yapın:

0 İyi

1 Biraz suçluluk hissiyle

2 Büyük bir suçluluk hissiyle

3 Çok büyük bir suçluluk hissiyle

9) Son 3 ayda cinsel ilişki arzusunda artma veya azalma oldu mu?

Arzunuz geçmişe kıyasla arttı mı yoksa azaldı mı?

Hastanın arzusu değişmemiş veya artmışsa 0 puan verin; arzusu azalmışsa 1 puan verin.

0 Değişmeyen veya artan arzu

1 Azalan arzu

10) Ejakülasyon hacminde bir azalma fark ettiniz mi?

Hasta ejakülasyon (meni) hacminde herhangi bir değişiklik fark etmediyse 0 puan verin; ejakülasyon hacminin biraz azalmış olduğu hissine kapılmışsa 1 puan verin; ejakülasyon hacmi belirgin şekilde azalmışsa 2 puan verin; hiç ejakülasyon olmuyorsa 3 puan verin.

0 Değişiklik yok

1 Biraz azalmış

2 Belirgin şekilde azalmış

3 Ejakülasyon yok

11) Son 3 ayda cinsel ilişki sırasında ejakülasyon/boşalma (veya orgazma ulaşmak) zor oldu mu?

Penetrasyonla (cinsel birleşme/duhul) cinsel ilişki sırasında ejakülasyon yapabiliyor musunuz yoksa sadece partnerinizin manuel veya oral uyarısı ile mi ejakülasyon yapabiliyorsunuz?

Hasta ejakülasyonda zorluk yaşadığını bildirmiyorsa veya nadir durumlarda ejakülasyon veya cinsel doyuma, bir partner olmadan sadece otoerotik aktivite ile ulaşılabiliyorsa ya da hiç ulaşılamıyorsa 0 puan verin. Ejakülasyon ve cinsel doyuma ulaşma hala mümkünse ancak önemli bir çaba gerektiriyorsa, uzun süreli cinsel ilişki gerektiriyorsa veya bir partnerin varlığında bile sadece otoerotik aktivite yoluyla gerçekleşiyorsa ancak cinsel birleşme sırasında gerçekleşmiyorsa 1 puan verin.

0 Hayır

1 Evet

12) Kaç kilosunuz ve boyunuz ne kadar?

Hasta kilosu ve boyu sorulduktan sonra görüşmeci, hesaplama fonksiyonuna (vücut kitle indeksi = kilo [kg] / boy [m^2]) göre uygun bir puanlama yapmalıdır.

0 <25 kg/m^2

1 25–29.9 kg/m^2

2 30–34.9 kg/m^2

3 >34.9 kg/m^2
